# Monitoring Cyanobacterial Blooms during the COVID-19 Pandemic in Campania, Italy: The Case of Lake Avernus

**DOI:** 10.3390/toxins13070471

**Published:** 2021-07-08

**Authors:** Roberta Teta, Gerardo Della Sala, Germana Esposito, Mariano Stornaiuolo, Silvia Scarpato, Marco Casazza, Aniello Anastasio, Massimiliano Lega, Valeria Costantino

**Affiliations:** 1 Department of Pharmacy, University of Naples Federico II, Via Domenico Montesano 49, 80131 Napoli, Italy; roberta.teta@unina.it (R.T.); germana.esposito@unina.it (G.E.); mariano.stornaiuolo@unina.it (M.S.); silvia.scarpato@unina.it (S.S.); 2 Department of Marine Biotechnology, Stazione Zoologica Anton Dohrn, Villa Comunale, 80121 Napoli, Italy; gerardo.dellasala@szn.it; 3 Department of Sciences and Technologies, University of Naples Parthenope, Centro Direzionale di Napoli, Isola C 4, 80143 Napoli, Italy; marco.casazza@uniparthenope.it; 4 Department of Veterinary Medicine and Animal Production, University of Naples Federico II, Via Federico Delpino 1, 80137 Napoli, Italy; aniello.anastasio@unina.it; 5 Department of Engineering, University of Naples Parthenope, Centro Direzionale di Napoli, Isola C 4, 80143 Napoli, Italy

**Keywords:** cyanobacteria, cyanotoxins, water quality, cyanobacterial bloom, FDS, microcystins, cytotoxicity, remote sensing

## Abstract

Cyanobacteria are ubiquitous photosynthetic microorganisms considered as important contributors to the formation of Earth’s atmosphere and to the process of nitrogen fixation. However, they are also frequently associated with toxic blooms, named cyanobacterial harmful algal blooms (cyanoHABs). This paper reports on an unusual out-of-season cyanoHAB and its dynamics during the COVID-19 pandemic, in Lake Avernus, South Italy. Fast detection strategy (FDS) was used to assess this phenomenon, through the integration of satellite imagery and biomolecular investigation of the environmental samples. Data obtained unveiled a widespread *Microcystis* sp. bloom in February 2020 (i.e., winter season in Italy), which completely disappeared at the end of the following COVID-19 lockdown, when almost all urban activities were suspended. Due to potential harmfulness of cyanoHABs, crude extracts from the “winter bloom” were evaluated for their cytotoxicity in two different human cell lines, namely normal dermal fibroblasts (NHDF) and breast adenocarcinoma cells (MCF-7). The chloroform extract was shown to exert the highest cytotoxic activity, which has been correlated to the presence of cyanotoxins, i.e., microcystins, micropeptins, anabaenopeptins, and aeruginopeptins, detected by molecular networking analysis of liquid chromatography tandem mass spectrometry (LC-MS/MS) data.

## 1. Introduction

**Figure 1:**
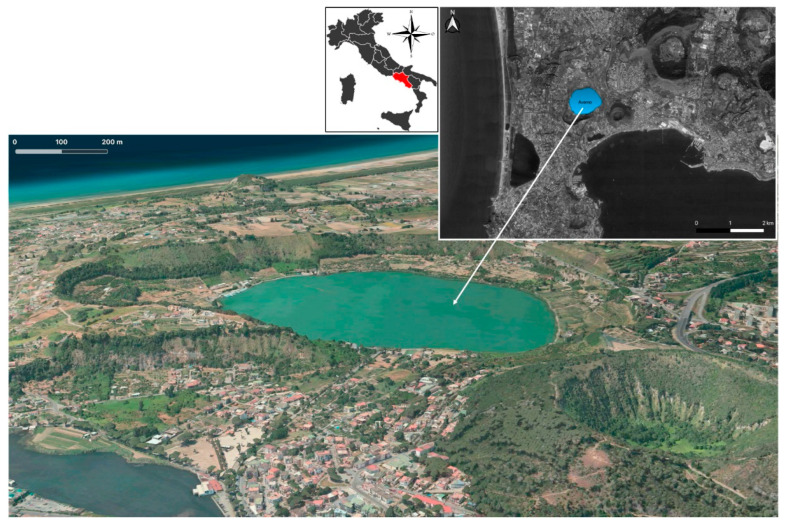
Lake Avernus: position and 3D view that reveals its volcanic nature; it is possible to recognize the shape of the volcanic crater that the lake fills.

Lake Avernus (Lago d’Averno) is a volcanic lake located in the area known as Phlegrean Fields (*Campi Flegrei*), 2.5 mi west of Pozzuoli, in Campania, southern Italy (Figure 1). The area is known since the Romans, who settled its shores, on which villas and vineyards were built. Nowadays, the area has a strong anthropogenic pressure related to the permanent activities (agricultural activities, housing, etc.) and the recreational activities on the banks [1].

Our group is on the lookout for cyanobacteria bloom as part of a new time-efficient environmental monitoring strategy that uses satellite imaging to guide in situ sampling in selected spots, followed by lab analyses, i.e., microscopy observations and chemical analyses [2,3].

Cyanobacteria harmful algal blooms (cyanoHABs) are triggered by an influx of nutrients, which can occur—for example—through urbanization or agricultural activities [4] as well as environmental conditions, such as warming temperatures. Cyanobacteria blooms have emerged as one of the major issues in the world because they produce cyanotoxins, mostly peptide molecules, which are toxic for humans and animals. Among others, microcystins (MCs) are the most studied [5,6] and comprise more than 100 congeners. Microcystins are bioaccumulated through the trophic chain in fish, mussels, and crustaceans, representing a major concern for food safety. A recent review [7] pointed out new evidence on the nephrotoxicity of MCs, aside from the liver.

Lake Avernus experienced a cyanobacterial bloom in 2008 [8]; in particular, Ferranti et al. described a bloom of *Planktothrix rubescens* and identified anabaenopeptin B and anabaenopeptin F from samples collected between March and June 2007 as the major toxins on the basis of mass spectrometry data.

Here we report on the cyanobacterial bloom that occurred in Lake Avernus at the turn of the COVID-19 pandemic lockdown in Italy (from 6 March to 15 June 2020). This study started with the detection through satellite data processing of an out-of-season cyanoHAB. Sampling was performed on 11 February 2020 and microscopic analysis unveiled water samples to be mainly composed of *Microcystis* sp. Analyses of the temporal progression of the bloom highlighted that this unusual “winter bloom” completely disappeared at the end of COVID-19 lockdown, presumably as a result of the closure of almost all urban activities, thus suggesting an anthropogenic origin.

To assess potential harmfulness of the *Microcystis* bloom, crude extracts from collected samples were evaluated in vitro for their cytotoxicity in two different human cell lines, namely normal dermal fibroblasts (NHDF) and breast adenocarcinoma cells (MCF-7). The highest antiproliferative activity was shown to be exerted by the chloroform extract, whose dereplication unveiled the presence of toxic cyanopeptides, including MCs, micropeptins, anabaenopeptins, and aeruginopeptins. Metagenomic detection of the *mcyB* gene from the MC biosynthetic gene cluster confirmed the bloom forming *Microcystis* sp. to produce MCs.

## 2. Results

### 2.1. Remote and Proximal Sensing Analysis

The continuous analysis of the multispectral data acquired by Sentinel 2 satellite, downloaded monthly, allowed us to identify an anomalous bloom of cyanobacteria on the shores of Lake Avernus during the month of February 2020.

The analysis of satellite data allowed for the reconstruction of the bloom’s temporal evolution and the distribution of cyanobacteria along the shores of the lake and, therefore, allowed us to define the water sampling spots.

Concerning the temporal analysis of satellite imageries, the anomaly of the presence of the bloom in winter—when, normally, due to the cold temperature, the blooming conditions are not optimal—was immediately evident.

**Figure 2:**
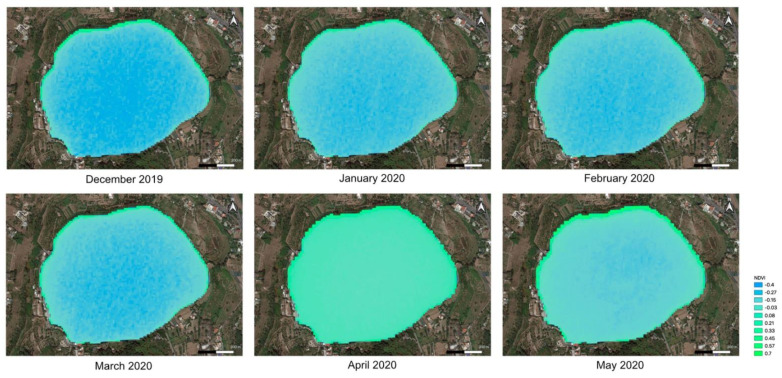
Temporal sequence from December 2019 to May 2020 of the processing of satellite multispectral data; in particular, the rendering of the NDVI index in false color maps is reported. The dataset used is that of Sentinel 2 and the spatial resolution is 10 m/px.

The temporal trend was obtained through the satellite data-processing sequence; specifically, the NDVI index observed between December 2019 and February 2020 was rendered in false color maps (Figure 2). Combining data registered by the Italian Local Health authority through reports from inhabitants of that area with visible inspections and rendered satellite data by radiometric analysis, the bloom onset was dated in February 2020. From the starting month, remote sensing analysis registered a continuous increment and spreading of chlorophyll reaching a maximum in April 2020, and then the trend was reversed.

**Figure 3:**
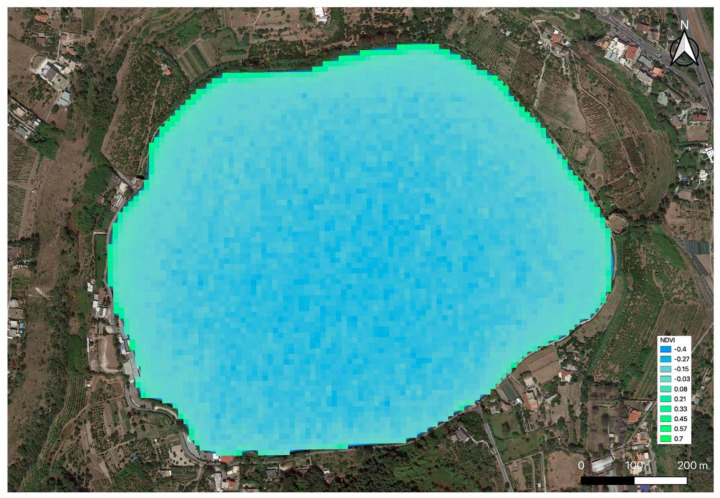
Enlargement of NDVI false color map of Lake Avernus dated 11 February 2020.

**Figure 4:**
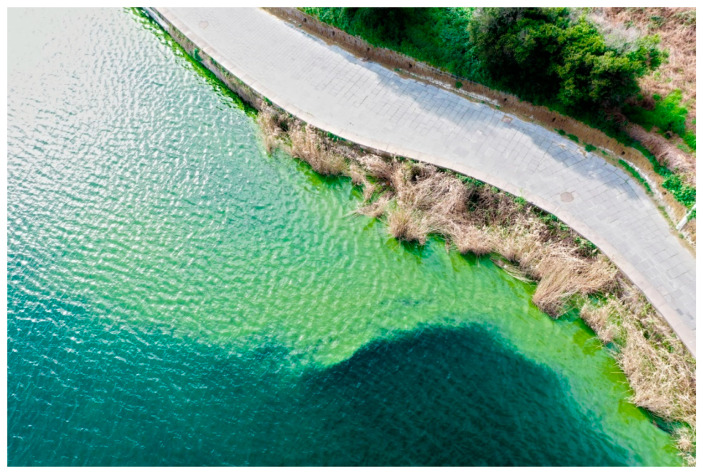
Image acquired during the drone flight performed on 11 February 2020. The scanned area was centered on lake shores where the blooming was most widespread as indicated by NDVI data and selected as the sampling target.

**Figure 5:**
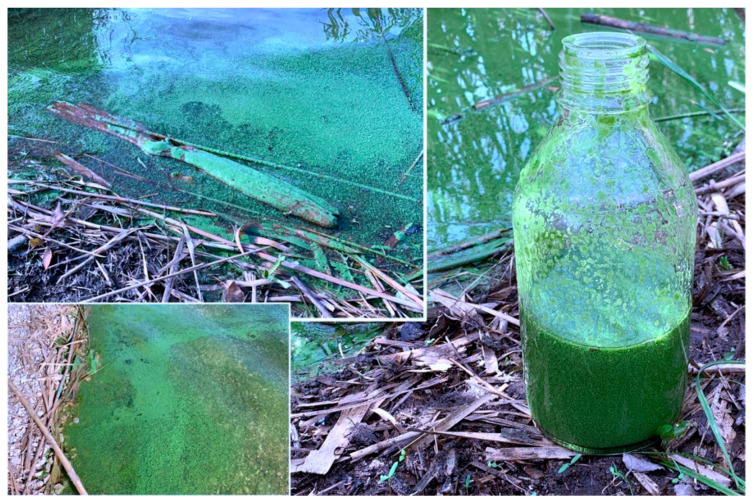
Images acquired during sampling carried out on 11 February 2020. All the sampling points were defined within the hierarchical process started using satellites and drones.

Regarding the spatial analysis, even though the phenomenon covers a large part of the shores of the lake over time, the points of origin/accumulation were not clearly recognizable on the NDVI map (Figure 3). Therefore, a survey of the area by a mini-drone equipped with specific payloads (see Section 4.1) was needed, and thanks to the improved resolution [9], it allowed for the localization of the sampling target spot (Figure 4) and for simultaneous remote acquisition and sampling on the site (Figure 5).

### 2.2. Sampling and Morphological Identification

Measurements of nitrates (0.5 mg/L) and phosphates (0.82 mg/L) on the collected sample revealed levels far above the European average annual concentrations, according to the European Environmental Agency (EEA) [10].

**Figure 6:**
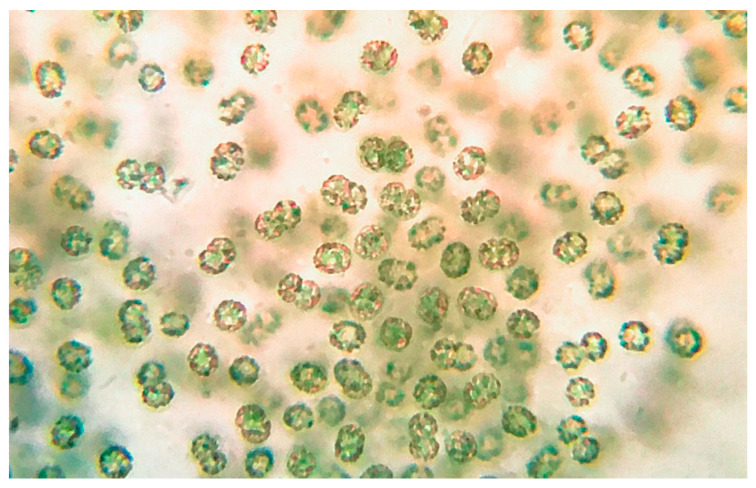
*Microcystis* sp. from cyanobacterial bloom at Lake Avernus.

Microscopic observation of the raw buoyant cell suspension (Figure 6) allowed us to distinguish cyanobacterial cells with morphological traits typical for *Microcystis* sp. such as spherical cells (2–8 µm) with dark-colored gas-filled vesicles, irregularly covered by colorless, mucilaginous sheath.

### 2.3. Chemical Analysis of the Bloom

**Figure 7:**
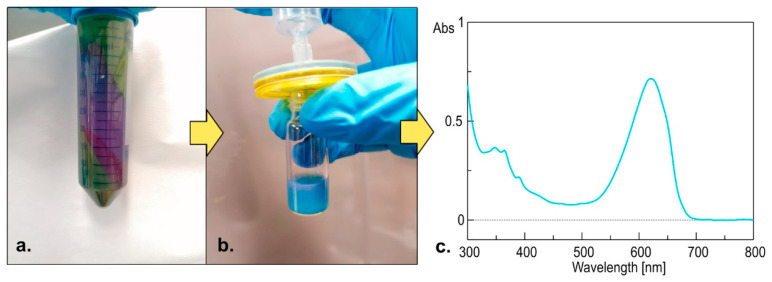
(**a**) Centrifugation of the cellular suspension, (**b**) filtration, and (**c**) UV/Vis analysis of the supernatant indicated the presence of phycocyanin in the sample, a typical cyanobacterial pigment.

The sample was centrifuged and pellet and supernatant were treated separately. The blue color of the supernatant was suggestive of the presence of the phycocyanin pigment that was confirmed by UV/Vis analysis (−λ_MAX_ = 260 nm) of a filtered aliquot of the sample (Figure 7).

The supernatant was partitioned with BuOH while the pellet was extracted with MeOH and CHCl_3_ mixtures. The organic extracts were tested for their cytotoxic activities (Section 2.4) and, to verify the production of MCs, the presence of their biosynthetic gene cluster was ascertained through quantitative PCR experiments on environmental DNA (Section 2.5).

The metabolic profile of the active chloroform extract was achieved through molecular networking of liquid chromatography high-resolution tandem mass spectrometry (LC-HR-MS/MS) data, obtained using an LTQ Orbitrap instrument with an electrospray (ESI) source. Data-dependent acquisition was used to trigger only MS^2^ scans in the mass range of 700–1500 amu, in which most of the molecular masses of known peptide toxins fall. The raw LC-MS data were pre-processed using the MZmine program [11], the .mgf MS2 data file generated by MZmine was submitted to the online platform at the Global Natural Products Social Molecular Networking website [12], and a feature-based molecular network was then created [13]. In order to refine the dereplication of known peptidic natural compounds (NP) in the extract, the Dereplicator tool was used in its variant, the Dereplicator+ [14], also able to annotate non-peptidic NP. In silico structure prediction coming from the Dereplicator+ was integrated with the chemical structural information deriving from matching with the GNPS library and with the information obtained through MolNetEnhancer [15] workflow, which allowed us to recognize the most abundant chemical classes per molecular cluster in the network.

**Figure 8:**
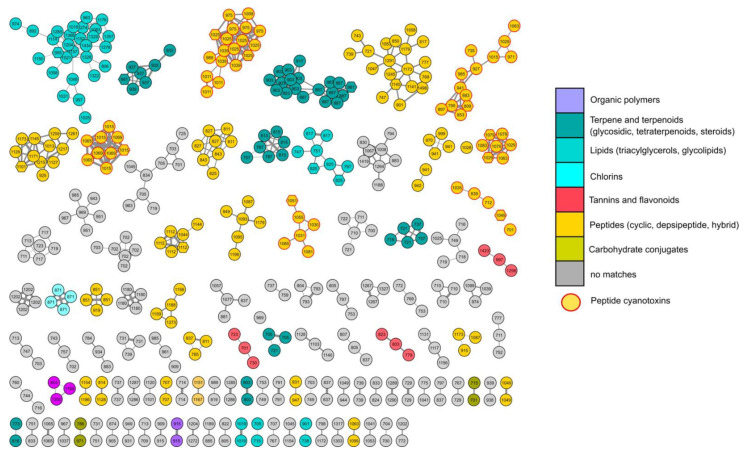
Enhanced molecular network of Lake Avernus’ cytotoxic extract. Nodes are labeled with parent mass. The color of each node is related to the compound classification derived from the MolNetEnhancer and nodes belonging to cyanotoxins are bordered in red. Nodes annotated by the Dereplicator+ with a putatively identified peptide cyanotoxin are represented as hexagons. Edge thickness is related to cosine score similarity.

The resulting enhanced network was then visualized using Cytoscape 3.8.2 [16]. In the network (Figure 8), the color of each node is mapped to the compound classification derived from the MolNetEnhancer. The network is composed of 435 nodes grouped into 107 clusters, 41 of which belonging to 6 recognized chemical classes of compounds, and the rest are unknown features. Nodes annotated by the Dereplicator+ (Table S1) with a putatively identified peptide cyanotoxin are represented as hexagons.

As expected from the mass range, the most abundant class of compounds in the active extract is represented by peptides, comprising cyclic, hybrid peptides, and depsipeptides. Six clusters revealed the presence of peptide cyanotoxins in the extract, which account for its toxicity (Table 1). Specifically, three nodes were annotated as microcystins (MC-LW, MC-M(O)R, [d-Asp]^3^MC-FR) [17], two belong to micropeptins (MP-88B [18], MP-T1 [19]), and other two were dereplicated as anabaenopeptin C [20] and aeruginopeptin 228B [21]. Nodes in the cyanotoxin clusters, which could not be associated to any known compound, indicated the presence of new molecular entities, structurally related to the cyanotoxins, which require further studies for their identification.

### 2.4. In Vitro Cytotoxicity of Organic Extracts from the Cyanobacterial Bloom in Lake Avernus

**Figure 9:**
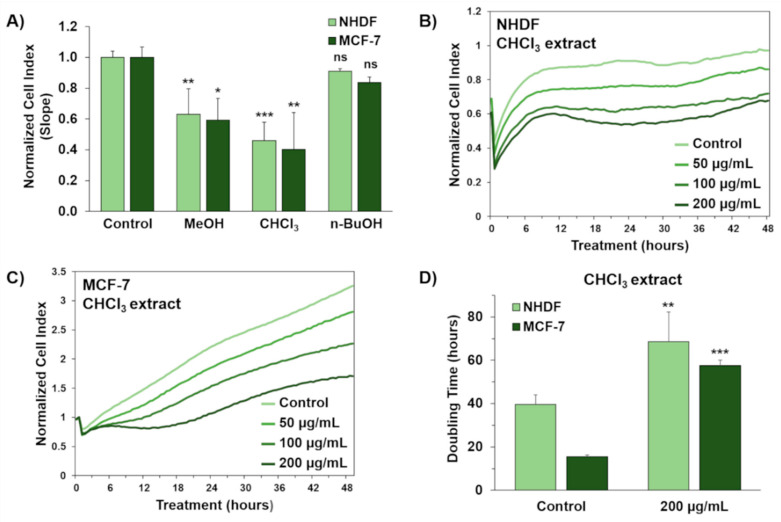
In vitro cytotoxicity of organic extracts from the cyanobacterial bloom in Lake Avernus. Antiproliferative effects of methanol (MeOH), chloroform (CHCl_3_), and *n*-butanol (*n*-BuOH) extracts from the cyanobacterial bloom were evaluated in normal human dermal fibroblasts (NHDF) and breast adenocarcinoma cells (MCF-7). Cell growth was monitored in real time by using the xCELLigence system real-time cell analyzer. (**A**) Evaluation of cytotoxic activity of organic extracts at the highest concentration used in this study (200 μg/mL). Cytotoxic effects are reported as slope of normalized cell index (NCI) to describe the changing rate of growth curves after 24 h exposure to the crude extracts. NCI slope values are relative to controls treated with 0.5% DMSO vehicle. (**B**) Representative NCI traces of NHDF and (**C**) MCF-7 cells after exposure to 0.5% DMSO vehicle (control) and different concentrations (50, 100, and 200 μg/mL) of the CHCl_3_ extract from the cyanobacterial bloom. (**D**) NCI doubling times of NHDF and MCF-7 cells after 24 h exposure to 0.5% DMSO vehicle (control) and 200 μg/mL of the CHCl_3_ extract from the cyanobacterial bloom. Doubling time is the time required for cell index to double. Data are presented as mean ± SD; all experiments were performed at least in triplicate. The one-way analysis of variance (ANOVA) method was used to compare means of groups and Dunnett’s method was used as a post hoc test. Statistical significances are referred to the DMSO control. * *p* < 0.05; ** *p* < 0.01; *** *p* < 0.001; ns, not significant.

Organic extracts from the cyanobacterial bloom were assessed for their cytotoxicity in two different human cell lines, using dermal fibroblasts (NHDF) as a normal cell model and MCF-7 breast adenocarcinoma cells as a tumor cell model (Figure 9). Cell proliferation was monitored in real time through the xCELLigence cell analyzer, which converts electronic impedance alterations into a unitless parameter, called the normalized cell index (NCI), thus reflecting cell viability and morphology.

Methanol, chloroform, and *n*-butanol extracts from the cyanobacterial bloom were evaluated individually at three different concentrations (50, 100, and 200 μg/mL) for 48 h. The chloroform extract resulted to be the most toxic towards both NHDF and MCF-7 cells within the first 24 h of exposure. Indeed, the chloroform fraction was shown to elicit a significant reduction (approximately 60%) in the slope of the growth curve at the highest dose tested, with a concomitant increase in cell doubling time, as compared to the relevant controls treated with 0.5% DMSO vehicle (Figure 9).

In the light of these findings, the chloroform fraction is argued to be enriched with putative cytotoxic cyanobacterial metabolites.

### 2.5. Metagenomic Detection of the mcy Gene

**Figure 10:**
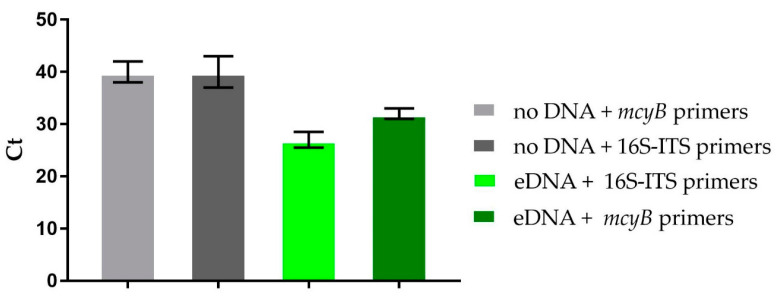
Presence of the *mcyB* gene in the cyanobacterial sample collected in Lake Avernus. qPCR results showing the primer-specific amplification of the *mcyB* gene as a function of the threshold PCR cycle (Ct). The amplification of 16S-ITS is shown as positive control of amplification. No amplification could be detected in the absence of eDNA (Ct > 38).

To confirm the presence of MCs producing cyanobacteria in the collected bloom, we tested environmental DNA for the presence of the *mcyB* gene from the MC biosynthetic gene cluster. Metagenomic DNA was extracted as reported [5] and analyzed by quantitative PCR, using SYBR Green I technology and *mcyB*-specific primers (see Section 4.4). The cyanobacterial 16Sinternal transcribed spacer (16S-ITS) rRNA gene (here used as housekeeping gene) appeared at the 27th cycle of PCR amplification (Figure 10), confirming the quality of the extracted DNA, while *mcyB* amplicon appeared at the 32th cycle, proving the presence of the gene in the collected bloom sample. In the absence of sample DNA, no amplification products could be detected, confirming the specificity of the amplification reaction.

## 3. Discussion

Abnormal environmental phenomena are often related to human actions and their study can detect correlations that would otherwise be difficult to discover. During our continuous monitoring of the Campania’s coasts and inland lakes and basins, we discovered the presence of an out-of-season *Microcystis* sp. harmful bloom event along the shores of Lake Avernus.

Most of cyanobacterial HABs are known to develop in areas with warm climates under a combination of factors such as nutrients (P, N), sunlight for photosynthesis, and water pH [22]. The bloom arose on Lake Avernus unexpectedly in February 2020, in the middle of the winter season in Italy. The environmental triggers were investigated and resultingly ascribed to an unusual chain of meteorological events that occurred at that time in Italy. In detail, an anomalous heatwave was registered in February 2020, not only in Italy, but also in many other countries, so that February 2020 has been defined Earth’s 2nd warmest February and 3rd warmest month on record. The weather conditions stimulated early and abundant vegetation flowering in the area surrounding Lake Avernus. The following heavy precipitations washed away nutrients, fertilizers, and pesticides from the cultivated fields to the lake. This excessive nutrient loading caused an overproduction of vegetation (eutrophication) in the lake and, consequently, the harmful cyanobacterial blooming.

The temporal course of the bloom was monitored and showed the highest peak at the turn of the COVID-19 lockdown in Italy, which started on 6 March 2020, and a sudden stop at the end of the lockdown, suggesting a relationship with the concomitant variation of anthropogenic activities. This can be explained in that the stop of all the agricultural, commercial, and recreational activities on the lake suddenly eliminated the continuous supply of nutrients to lake, normally derived from incorrect behaviors and also from the simple washout of fertilizers to the lake, for example.

The early detection, identification, and monitoring of the environmental problem was successfully achieved through FDS [2,3]. FDS is a new multidisciplinary strategy for monitoring wide coastal areas in a short time, avoiding numerous sampling procedures and analyses, and reducing detection time and costs. The strategy is the result of the study and application of different approaches for the monitoring of cyanobacteria and their toxins and combines the advantages of each of them.

The bloom was detected early using data acquired by the Sentinel 2 satellite. The NDVI index together with information coming from the mini-drone allowed us to localize the bloom and start the collection’s campaign.

The microscopic observation of the raw buoyant cell suspension revealed cyanobacterial cells with morphological features typical for *Microcystis* sp. As *Microcystis* is known as a toxin-producing strain, a preliminary metagenomic screen of the bloom sample was carried out for detection of microcystin biosynthetic genes. PCR amplification of a *mcyB* fragment suggested the presence of the microcystin synthetase gene cluster in the environmental DNA sample. However, PCR detection of this *mcy* fragment is not the proof for the production of the encoded metabolite, in that, in some cases, biosynthetic genes remain phenotypically cryptic unless exposed to specific environmental conditions [23]. Microcystin production was confirmed through chemical analyses and cytotoxicity assays, thus unveiling potential harmfulness of this out-of-season cyanoHAB.

Specifically, the chloroform extract displayed a similar extent of toxicity towards two human cell lines, namely normal dermal fibroblasts (NHDF) and breast adenocarcinoma cells (MCF-7), at the highest dose tested (200 μg/mL). Therefore, the chloroform extract was subjected to LC-HRMS/MS analyses and molecular networking. The new tools (Dereplicator+ and MolNet Enhancer) available on GNPS for dereplication and molecular networking allowed us to reveal the presence of a series of peptide cyanotoxins, such as microcystins, micropeptins, and anabaenopeptins, which may be responsible for the toxicity of the extract. In addition to known cyanotoxins, several unknown structural congeners were revealed (work in progress to elucidate them).

The out-of-season *Microcystis*’ bloom in Lake Avernus is unprecedented in the literature and is of great interest, both for the risks associated with toxicity for people, animals, and ecosystems in general, and also because the lake is directly connected to the sea, which it pours into and can thus generate more extensive coastal marine phenomena. The nearby coastal area is indeed dotted with similar coastal lakes where the toxic blooms can also arise; thus, the contamination can spread throughout the entire coastal strip. In addition, several shellfish farms are settled in those lakes and coastal area; therefore, their eventual contamination can affect the food chain with severe consequences. Our multidisciplinary approach once again proved successful in the early detection of the environmental problem and in its identification and progression.

## 4. Materials and Methods

### 4.1. Remote and Proximal Sensing Analysis

In recent years, the scientific community has increasingly used satellite data for the calculation of biophysical parameters of the observed areas. Among satellites, Landsat 8 and Sentinel-2 are commonly used for environmental studies. In particular, the availability of multispectral data allows for the calculation of specific indices that have proved to be particularly useful and reliable [24].

The best known and most tested multispectral index is certainly the NDVI, calculated through a combination of red and near infrared (NIR) bands, producing values between −1 and 1. The expression to calculate NDVI is [25,26]:

(1) $$\mathrm{NDVI}=\frac{\mathrm{NIR}-\mathrm{red}}{\mathrm{NIR}+\mathrm{red}}$$

where NIR and red refer to the reflectance values for the specified satellite bands. Sentinel-2, operated by the European Space Agency (ESA), is constituted by a group of two polar satellites operating in the same orbit, phased at 180° to each other. Its swath width is 290 km and its revisit time is 10 days (at the equator, 1 satellite) or 5 days (2 satellites, cloud-free conditions, equivalent to 2–3 days at mid-latitudes). Sentinel-2 carries an optical instrument payload, the multispectral instrument (MSI), that with a push-broom concept samples 13 spectral bands: 4 bands at 10 m, 6 bands at 20 m, and 3 bands at 60 m spatial resolution. In the case of Sentinel-2, the red band corresponds to the fourth channel (bandwidth: 640–690 nm), while the NIR band corresponds to the eighth channel (bandwidth: 780–910 nm), with a pixel spatial resolution of 10 m. The same index can be calculated using band 5 (NIR) and band 4 (red). However, the pixel spatial resolution is of 30 m. This is why, in order to obtain a higher spatial resolution, Sentinel-2 data were used instead of Landsat 8. In addition to the lower spatial resolution, the rendering of the Landsat 8 data presented several problems due to the high cloud cover in the acquisition dates and a high “banding” that did not allow us to correctly examine the phenomenon.

Several other indices (CI, MCI, CARI, Chlgreen, Chlred-edge, RBD, and CVI) could also be calculated on the basis of available satellite data having specific available spectral bands to formulate specialized indices that allow researchers to carry out not only qualitative, but also quantitative analyses.

Among the others, the MODerate resolution imaging spectroradiometer (MODIS), installed on both the Terra and Aqua satellites, and providing imagery of 36 bands at three spatial resolutions (from 250 m to 1000 m), allows researchers to develop an algorithm specifically designed to quantify the concentration of Chlorophyll-a. [27] Chlorophyll-a near-surface concentrations, expressed in mg/m^3^, are obtained with a pixel spatial resolution of 1 km x 1 km with a daily temporal resolution.

The limited resolution of most of these products of these platforms reduce the study of only large-scale phenomena. The combined use of remote (satellites and airplanes) and proximal (drones) sensing techniques has exceeded the previous limits allowing researchers to reach new goals in earth observation.

The purpose of remote/proximal sensing is related to the early detection of blooms; thus, this study focuses on qualitative detection methods, which provide a reliable first screening of bloom phenomena. In particular, the application of these observation techniques was to detect the presence of anomalies and to circumscribe the areas on which to perform further investigations and samplings [28]. This is why the NDVI index mapping was sufficient to meet the planned objective and, above all, to maximize the efficiency of the process in terms of time and costs.

NDVI maps were generated, on the basis of elaborated Sentinel-2 data, classifying the results on 10 distinct classes and representing them in a false-color scale, both customized for the specific problem and calibrated with field experiments. In this regard, ground truth points were defined, where the certainty of the presence of bloom was attributed. The entire analysis process was standardized in all steps, so that it can be replicated for each download of data associated with different dates. This approach made it possible to compare each processing and clearly visualize the trends.

Regarding the proximal sensing techniques, a mini-drone was used for the specific purpose. It carried two electro-optical sensors: a 12-megapixel multispectral camera able to acquire three bands (Orange + Cyan + NIR) and a 20 megapixels RGB camera with a one-inch sensor.

### 4.2. Sampling, Extraction, LC-MS, and Molecular Networking Analysis

The bloom sample (0.5 L) was collected in sterilized glass bottles along the Avernus lakeshore (40°50′20.4″ N 14°04′33.6″ E) on 11 February 2020. The raw cell suspension was immediately visualized in the mobile lab using an optical microscope combined with an OMAX 18 MP CMOS cooled camera and analyzed using ToupView software. Taxonomic classification and identification were performed according to Komarek et al. [29].

Environmental parameters were measured: temperature (air—Extech, water—TFA), salinity (optical refractometer MR100ATC), pH (pH meter—Hanna instrument), and nitrate and phosphate content (colorimeter—Hanna instrument).

The sample (0.3 L) was centrifuged (10,000 rpm for 5 min) to collect the suspended particles.

Once sonicated for 5 min, the pellet was then extracted with MeOH (100%, 0.3 L), MeOH/CHCl_3_ (1:1, 0.3 L), and CHCl_3_ (100%, 0.3 L). Chloroform extracts were combined, and all the extracts were paper filtered and concentrated in vacuo, yielding 851.8 mg of the MeOH extract and 302.8 mg of the chloroform extracts. Supernatant was extracted with BuOH. The BuOH phase was concentrated in vacuo affording to 109.6 mg of extract.

All the obtained extracts were subjected to cell viability assays. The active chloroform extract (Section 2.4 and Section 4.3) was re-suspended in MeOH (100%) at a concentration of 5 mg/mL. The sample was subjected to LC-HRMS and LC-HRMS/MS analyses. Experiments were performed using a Thermo LTQ Orbitrap XL high-resolution ESI mass spectrometer coupled to an Agilent model 1100 LC system, which included a solvent reservoir, in-line degasser, binary pump, and refrigerated autosampler. A 5-µm Kinetex C18 column (100 × 2.10 mm), maintained at room temperature, was eluted at 200 mL min^−1^ with H_2_O (supplemented with 0.1% HCOOH) and MeOH using gradient elution. The gradient program was as follows: 10% MeOH for 3 min, 10–100% MeOH for 30 min, and 100% MeOH for 7 min. Mass spectra were acquired in positive ion detection mode. MS parameters were a spray voltage of 5 kV, a capillary temperature of 285 °C, a sheath gas rate of 32 units N_2_ (ca. 150 mL/min), and an auxiliary gas rate of 15 units N_2_ (ca. 50 mL/min).

Data were collected in the data-dependent acquisition (DDA) mode, in which the first to the ninth most intense ions of a full-scan mass spectrum were subjected to high-resolution tandem mass spectrometry (HRMS/MS) analysis. The *m*/*z* range for data-dependent acquisition was set between 700 and 1500 amu. HRMS/MS scans were obtained for selected ions with CID fragmentation, an isolation width of 2.0, a normalized collision energy of 35, an activation Q of 0.250, and an activation time of 30 ms. Data were analyzed using Thermo Xcalibur software. Raw files were imported into MZmine 2.53 [11]. The mass detection was performed on raw data and exact masses with mass level 1 and centroided masses with mass level 2, by keeping the noise level at 1000. Chromatograms were built using an ADAP module [30] with a minimum height of 1000, and *m*/*z* tolerance of 0.01 (or 20 ppm). For the chromatogram deconvolution, the local minimum search algorithm was used with the following settings: chromatographic threshold = 5%, minimum retention time range = 0.20 min, minimum relative height = 30%, minimum absolute height = 1000, minimum ratio of the peak top/edge = 1.3, and peak duration range = 0.0–6.0 min.

Peak alignment was performed using the Join aligner algorithm (*m*/*z* tolerance at 0.02 (or 10 ppm), absolute RT tolerance at 0.5 min). [M+Na-H], [M+K-H], [M+Mg-2H], [M+NH_3_], [M-Na+NH_4_], and [M+1, ^13^C] adducts were filtered out by setting the maximum relative height at 100%. Peaks without associated MS/MS spectrum were finally filtered out from the peak list. Clustered data were then exported to .mgf file for GNPS, while chromatographic data including retention times, peak areas, and peak heights were exported to a .csv file. A molecular network [31] was generated on GNPS’ online platform [12], using the Metabolomics workflow with the following parameters: the parent mass tolerance and MS/MS fragment ion tolerance were set at 0.05 Da and 0.5 Da, respectively; the cosine score was set at above 0.6; and there were above 5 matched peaks. Spectra were retained only if the nodes appeared in each other’s respective top 10 most similar nodes. The spectra in the network were then searched against GNPS spectral libraries using a cosine score above 0.6 and at least 5 matched peaks. To enhance the results of annotations of peptidic and non-peptidic NP in the network, the Dereplicator+ tool [14] was used with the following parameters: precurson and fragment ions mass tolerance were set to 0.05 Da and 0.2 Da, respectively; the minimum score for significance was set to 4; and the fragmentation mode was set to 2-1-3.

Outputs from molecular networking and the Dereplicator+ were combined through the MolNetEnhancer [15] workflow, and the integrated network was visualized and analyzed by Cytoscape [16].

### 4.3. Cell Viability Assays

Cell viability assays were performed using the xCELLigence system real-time cell analyzer (ACEA Biosciences, San Diego, CA, USA), as previously reported [32]. NHDF and MCF-7 cells were seeded at a cell density of 5000 cells/well and 3000 cells/well, respectively. Cells were cultured in DMEM high glucose (4.5 g/L) medium, supplemented with 10% fetal bovine serum, penicillin–streptomycin (100 U/mL), and 2 mM L-glutamine. Approximately 24 h after seeding, the medium was removed and cells were treated with a medium containing 50, 100, and 200 μg/mL of organic extracts from the cyanobacterial bloom sample. Organic extracts were dissolved in DMSO to prepare 40 mg/mL stock solutions and further diluted in the culture medium to perform antiproliferative assays. In all experiments, the final concentration of DMSO did not exceed 0.5%, which was shown to be well tolerated with no observable toxic effects to cells. Cytotoxic effects of crude extracts are expressed either as (a) slope values of cell growth curves or as (b) cell doubling times. Growth curves were generated by measuring cell index variations. The cell index was normalized just before treatment and converted into a normalized cell index (NCI). NCI slopes, doubling times, and real-time NCI traces were calculated through the RTCA-integrated software (v2.0.0.1301, ACEA Biosciences, San Diego, CA, USA).

### 4.4. RT-PCR Experiments

Furthermore, 250 ng of environmental DNA was amplified by PCR in an Applied Bio-System StepOne Plus Real-Time Detector system (Thermo Fisher Scientific) with the fluorescent double-stranded DNA-binding dye SYBR Green (Thermo Fisher Scientific). PCR screening of metagenomic DNA was performed by using the 16S-ITS rRNA cyanobacterial specific primers CYA359F (5′-GGGGAATYTTCCGCAATGGG-3′) and 373R (5′-CTAACCACCTGAGCTAAT-3′), and the specific *mcyB* primers FAA (5′-CTATGTTATTTATACATCAGG-3′) and RAA (5′-CTCAGCTTAACTTGATTATC-3′), as previously reported [4]. Primers were used at 200 nM final concentration and designed to work under the following cycling conditions: 95 °C for 10 min, followed by 40 cycles at 95 °C for 15 s and 60 °C for 1 min. Samples were run in triplicate. The sample was considered positive for the presence of the *mcyB* gene when the amplification product appeared before the end of the exponential phase of the PCR.

## Figures and Tables

**Table 1:** Peptide cyanotoxins as annotated by the Dereplicator+ and molecular networking.

t_R_ (min)	*m*/*z*	Molecular Formula [M+H]^+^	GNPS’ Annotation ^1^
25.3	1015.5304	C_51_H_71_O_12_N_10_^+^	[d-Asp^3^] MC-FR
25.3	1025.5350	C_54_H_73_O_12_N_8_^+^	MC-LW
25.7	1029.5084	C_48_H_73_O_13_N_10_S^+^	MC-M(O)R
23.8	1051.5120	C_55_H_71_O_13_N_8_^+^	MP-T1
25.7	1079.5288	C_53_H_75_O_16_N_8_^+^	MP-88B
33.9	809.4596	C_41_H_61_O_9_N_8_^+^	AnaP-C
25.6	1049.5165	C_52_H_73_O_15_N_8_^+^	AerP-228B

^1^ Abbreviations: MC—microcystin; MP—micropeptin; AnaP—anabaenopeptin; AerP—aeruginopeptin.

## Data Availability

Not applicable.

## Supplementary Materials

The following are available online at https://www.mdpi.com/article/10.3390/toxins13070471/s1, Table S1: List of putatively identified metabolites by Dereplicator+, Figure S1: Extracted ion chromatograms of peptide cyanotoxins, Figure S2: MS/MS spectrum of putative [d-Asp^3^] MC-FR, Figure S3. MS/MS spectrum of putative MC-LW, Figure S4: MS/MS spectrum of putative MC-M(O)R, Figure S5: MS/MS spectrum of putative MP-T1, Figure S6: MS/MS spectrum of putative MP-88B, Figure S7: MS/MS spectrum of putative AnaP-C, Figure S8: MS/MS spectrum of putative AerP-228B.
