# Supplementary material for: Monitoring Cyanobacterial Blooms during the COVID-19 Pandemic in Campania, Italy: The Case of Lake Avernus
Source: Toxins (Basel). 2021 Jul 8;13(7):471. doi: 10.3390/toxins13070471 (PMC8310267; doi:10.3390/toxins13070471)
Supplement: Supplementary file 1 [file toxins-13-00471-s001.zip › toxins-1264444-supplementary.pdf]

toxins

Supplementary Materials: Monitoring Cyanobacterial Blooms during the COVID-19 Pandemic in Campania, Italy: The Case of Lake Avernus

Roberta Teta, Gerardo Della Sala, Germana Esposito, Mariano Stornaiuolo, Silvia Scarpato, Marco Casazza, Aniello Anastasio, Massimiliano Lega * and Valeria Costantino

Table S1. List of putatively identified metabolites by Dereplicator+ in Lake Avernus' active extract.

Dereplicator ID	Precursor Mass $[M+H]^+$	t_R
Paulomycin_E_Paulomycin_E	700.558	35.46
Leucomycin_V	702.398	18.89
Salarin_D_Salarin_D	703.299	16.28
2-(Dihydroxy-isopentenyl)-2'-isopentenyl-beta-carotin	705.579	15.50
2-(Dihydroxy-isopentenyl)-2'-isopentenyl-beta-carotin	705.58	37.79
Hirsutatin_A_4'-Methoxy	707.347	27.11
Piperazimycin_A_8-Deoxy	711.362	21.19
Aetheramide_A_Aetheramide_A	719.341	18.27
acyldepsipeptide_1	719.342	17.48
24-Nor-4(23)	721.609	37.04
24-Nor-4(23)	721.646	37.36
Geniposidic_acid_Me_ester	725.372	31.61
Geniposidic_acid_Me_ester	729.224	13.46
Amidepsine_F	730.256	11.45
PE(O-20:0/17:2(9Z	744.584	35.46
Carbamidocyclophane_M	749.331	20.07
PE(17:2(9Z	767.399	17.03
Antibiotic_RF_2691B_Antibiotic_RF_2691B	772.393	19.08
21-desethyl-21-cyclobutyl_spinosyn_D	772.531	31.43
Cycloartane-1	773.271	36.95
Carbamidocyclophane_S	784.233	5.20
CLWFTH	786.298	12.07
PG(O-20:0/18:3(9Z	787.581	38.30
34-Ethylhuratoxin_5-Deoxy	803.493	35.08
asteriidoside_H_sodium_salt	803.544	35.40
Indicanine_D_Indicanine_D	803.594	39.05
PE(P-20:0/22:6(4Z	804.578	36.35
PC(22:5(4Z	805.509	35.11
Anabaenopeptin_C_Anabaenopeptin_C	807.522	36.65
Anabaenopeptin_C_Anabaenopeptin_C	809.459	33.87
Ganosinensin_B	811.641	36.73
Ganosinensin_B	813.568	38.61
PG(O-20:0/20:4(5Z	813.597	38.51
PG(P-18:0/22:2(13Z	815.613	38.98
PG(P-18:0/22:2(13Z	815.659	39.06
PE(18:3(9Z	825.694	38.77

TG(15:0/20:4(8Z	825.694	38.15
TG(15:0/20:4(8Z	825.695	37.85
Pteratide_III_Pteratide_III	827.213	14.26
4''-O-Carbamoyl_ansamitocinoside_P-3	833.2	13.50
20:3-Glc-cholesterol_3-O-(6'-O-(11Z	837.592	35.41
Microsclerodermin_C_2''-Deoxy	850.436	17.89
Microsclerodermin_C_2''-Deoxy	851.357	22.75
Grividomycin-III	851.382	22.81
pheophytin_a	871.539	39.39
pheophytin_a | pheophytin-a	871.573	39.56
Glycerol_1	887.568	36.95
Glycerol_1	887.568	37.64
7-deformyl-7-hydroxymethyl-pheophytin_b/b'	887.569	37.14
(22S)-cholest-5-ene-1beta	887.57	36.08
(22S)-cholest-5-ene-1beta	887.57	35.44
TG(14:0/22:5(4Z)	901.726	37.30
Cycloartane-1	903.563	36.95
Bidenphytin_B_Bidenphytin_B	903.564	35.47
Bidenphytin_B_Bidenphytin_B	903.564	35.14
2-O-α-L-Rhamnopyranosyl-D-glucose_Hexadecyl_glycoside	915.669	37.03
TG(20:3(5Z	915.705	37.32
Somamide_B_Somamide_B	919.481	27.34
Pneumocandin_A0_5-Demethyl	931.594	39.33
Oleanolic_acid_bisdesmosides_3-O-[6-O-Decyl-β-D-glucuronopyranoside]	935.57	37.52
Pregnane-3	937.545	37.10
Ossamycin_28-Methyl	938.541	39.43
Didemnin_B	941.697	28.23
PI(20:1(11Z)/22:0)_1-(11Z-eicosenoyl)-2-docosanoyl-glycero-3-phospho-(1'-myo-inositol)	949.712	38.85
Fucoxanthinol_3-O-(9Z-Eicosenoyl)	951.567	36.01
Chlorobactene_1'	951.727	39.28
Chlorobactene_1'	951.728	39.83
TG(16:1(9Z)/22:5(4Z)	955.477	38.65
Lyngbyastatin_8_N-De-Ac	961.479	29.93
4-(2'-carboxyphenyl)-4-oxobutyryl-CoA	967.723	37.87
Alatogenin_3-O-[α-L-Rhamnopyranosyl-(1→2)-β-D-glucopyranoside]	974.51	32.77
5-cis-8-cis-Tetradecadienoyl-CoA	974.838	37.65
Alatogenin_3-O-[α--L-Rhamnopyranosyl-(1→2)- β -D-glucopyranoside]	975.553	25.35
Megalomycin_C2	975.596	25.38
Al(III)-acremonpeptide_D	985.732	37.83
mixirin_B	999.739	15.27
Spirosta-5	1009.5	25.57
Spirosta-5	1011.45	24.45
Discokiolide_B_Demethoxy	1011.59	24.42
[D-Asp3]-Microcystin-FR	1015.53	25.34
Microsclerodermin_A_46-Deoxy	1015.55	25.41

Pneumocandin_A0_1''	1015.55	32.71
Pneumocandin_A0_1''	1015.59	25.38
2-Amino-6-octadecene-1	1019.75	38.76
2-Amino-6-octadecene-1	1019.79	39.10
Microcystin_Microcystin_LW	1025.5	24.81
Microcystin_Microcystin_LW	1025.53	25.34
Microcystin-M(O)R	1029.49	25.68
Mixirin_C_Mixirin_C	1029.53	26.04
Cilofungin	1030.49	23.64
Pneumocandin_Bo	1037.6	33.14
Bacillomycin_D2	1039.47	25.63
TYLFTNS	1039.51	25.64
WS_9326A_2''S	1039.56	25.62
SJG-1	1044.55	21.79
Aeruginopeptin_228B	1049.46	23.71
Aeruginopeptin_228B	1049.51	25.62
bacillomycin_Lc_4	1049.54	25.62
Micropeptin_T1_Micropeptin_T1	1051.51	23.84
Pneumocandin_A0_5-Demethyl	1053.61	19.83
Subtulene_A_Subtulene_A	1055.51	23.47
Iturin_C_Iturin_C2	1057.72	35.36
Iturin_C_Iturin_C2	1063.58	20.78
Aquachelin-B	1065.49	24.81
Pyoverdin_G4RA_4'-Amide	1065.54	24.76
Pneumocandin_Bo	1065.55	25.05
Pneumocandin_A0_5-Demethyl	1065.57	24.88
Pompanopeptin_A_Pompanopeptin_A	1069.47	24.49
Kapakahine_C_Kapakahine_C	1069.52	24.44
Pompanopeptin_A_Pompanopeptin_A	1077.84	39.24
Micropeptin_88B_Micropeptin_88B	1079.52	25.68
KYNPCSNYL	1081.47	24.45
KYNPCSNYL	1083.54	25.28
hymenamide_J	1085.5	24.43
Prenylagaramide_A_Prenylagaramide_A	1087.58	33.15
KYNPCSNYL	1093.59	25.28
GlcNalpha1-6Ins-1-P-Cer(t18:0/26:0)_N-(hexacosanoyl)-4R-hydroxysphinganine-1-phospho-(1'-[2-amino-2-deoxy-D-glucopyranosyl-alpha1-6-D-myo-inositol])	1099.8	37.87
hymenamide_J	1101.75	35.35
Tripropeptin_Tripropeptin_Z	1103.86	38.87
Tripropeptin_Tripropeptin_Z	1112.54	28.25
Didemnin_B_Stereoisomer(?)	1112.62	28.19
Didemnin_B	1112.66	28.23
Massetolides_Massetolide_E	1112.71	28.25
Glycerol_1	1117.59	32.75
Pyoverdin_PL8_Pyoverdin_PL8	1127.77	35.55
Plusbacin_A_10-Deoxy	1128.61	23.99
Didemnin_B_Stereoisomer(?)	1129.78	36.35
Tripropeptin_Tripropeptin_B	1140.61	26.98

Plusbacin_A_10-Deoxy	1151.85	39.19
Tripropeptin_Tripropeptin_B	1156.61	26.89
Neopeptin_Neopeptin_C	1173.81	36.36
VQVEYKGETK	1179.54	33.22
VQVEYKGETK	1180.66	31.68
Linearmycin_C	1180.71	31.66
N/A	1180.77	31.66
Leucinostatin_B2	1186.8	36.11
Sandramycin_Dideoxy	1189.58	33.15
Polymyxin_E	1199.68	37.83
Leucinostatin_B2	1200.19	31.66
M(IP)2C(t18:0/16:0)_N-(hexadecanoyl)-4R-hydroxysphinganine-1-O-[myo-inositol-1-phosphoryl-6-D-mannopyranosyl-alpha1-2-myo-inositol-1-phosphate]	1202.64	31.69
HPG-8	1202.7	31.66
Antibiotic_GE_2270_8	1287.16	28.28
Antibiotic_GE_2270_8	1298.39	15.46
3-Hydroxyspirostan-12-one_3-O-[α-L-Rhamnopyranosyl-(1→3)-β-D-xylopyranosyl-(1→2)-[β-D-xylopyranosyl-(1→3)-β-D-glucopyranosyl-(1→3)]-β-D-glucopyranosyl-(1→4)-β-D-galactopyranoside]	1327.57	35.82
GM-I	1423.59	13.34

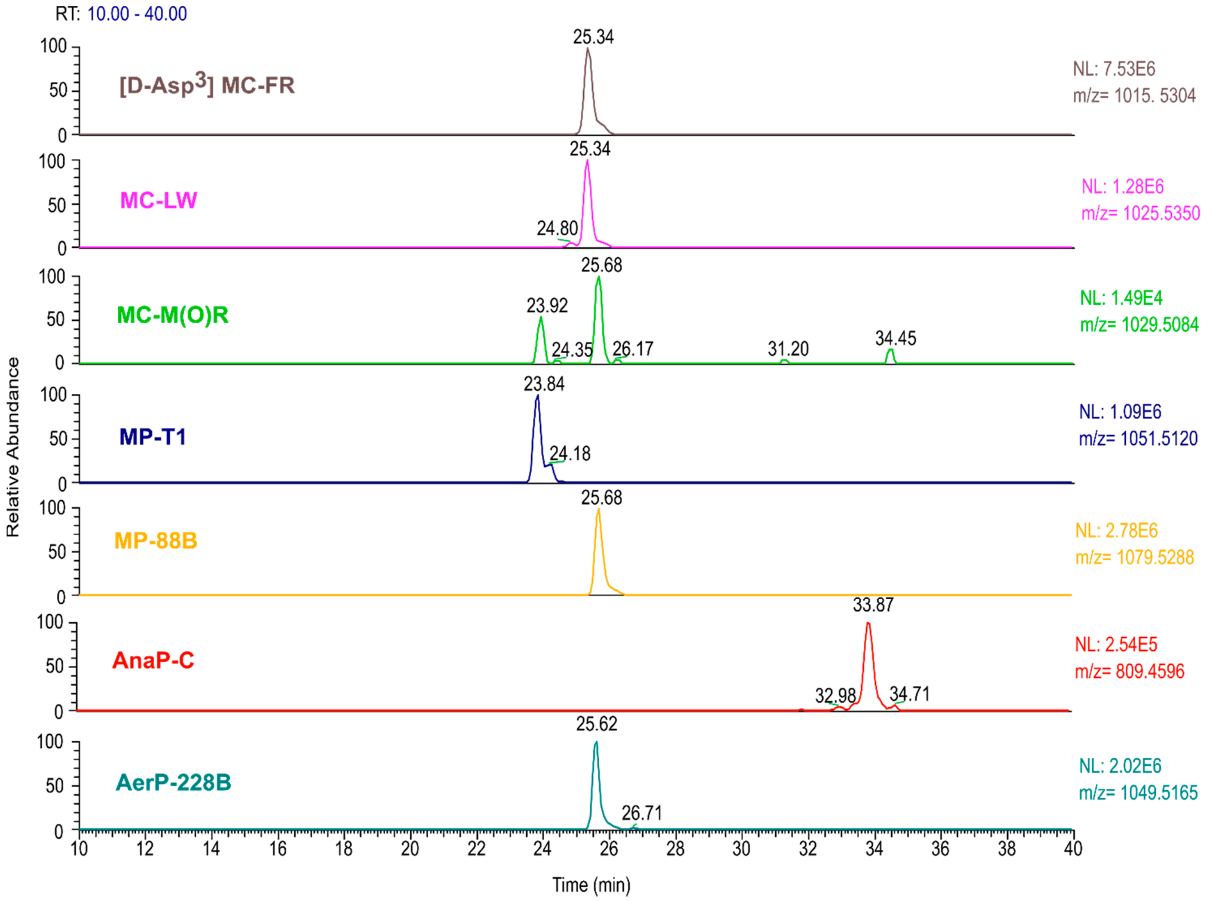

Figure S1. Extracted ion chromatograms of the putatively identified peptide cyanotoxins at *m/z* 1015.5304 ([D-Asp[3]] MC-FR), *m/z* 1025.5350 (MC-LW), *m/z* 1029.5084 (MC-M(O)R), *m/z* 1051.5120 (MP-T1), *m/z* 1079.5288 (MP-88B), *m/z* 809.4596 (AnaP-C), *m/z* 1049.5165 (AerP-228B).

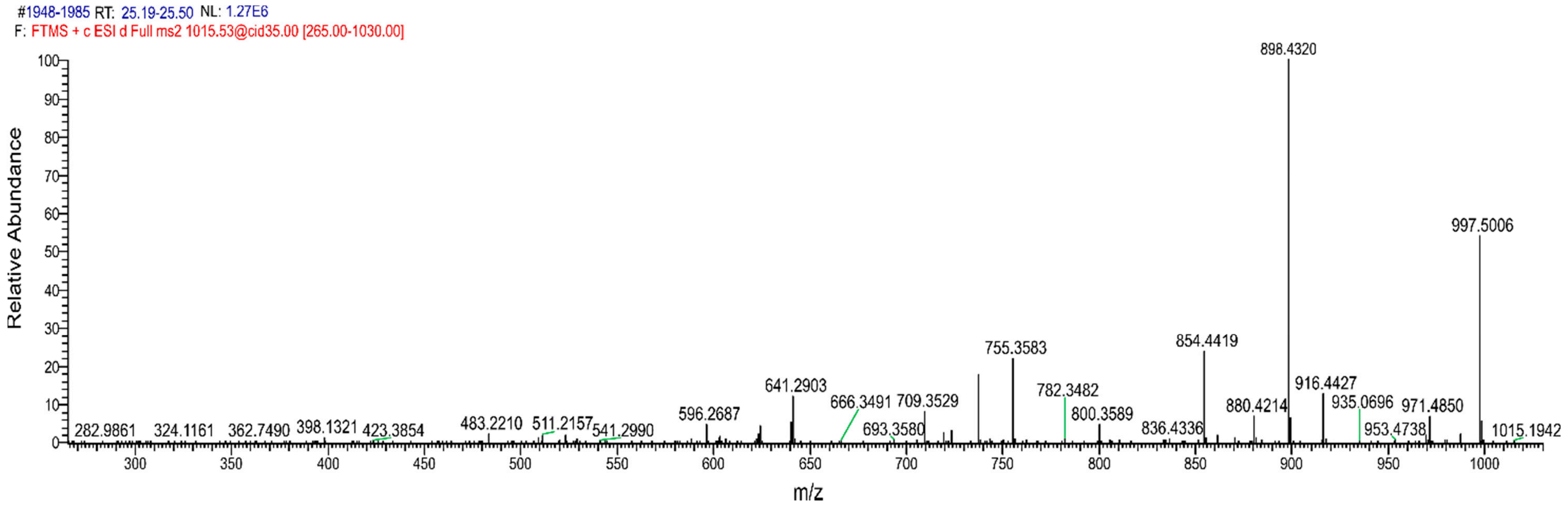

Figure S2. High resolution MS/MS spectrum of the $[M+H]^+$ ion at *m/z* 1015.5304, putatively identified as [D-Asp[3]] MC-FR.

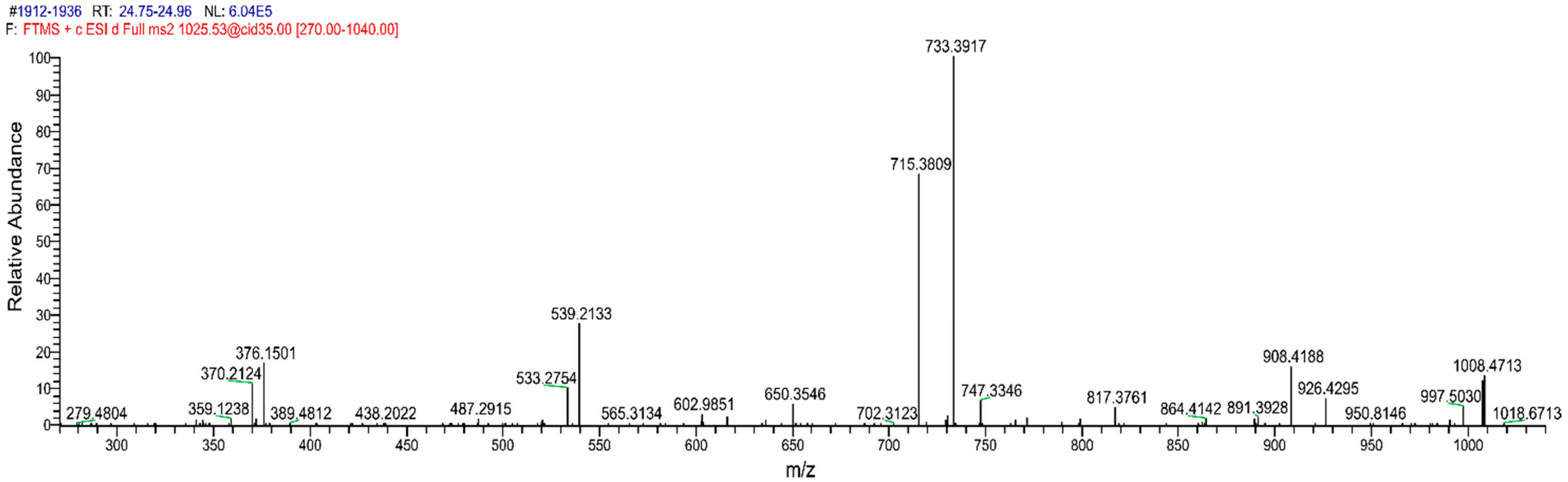

Figure S3. High resolution MS/MS spectrum of the $[M+H]^+$ ion at *m*/*z* 1025.5350, putatively identified as MC-LW.

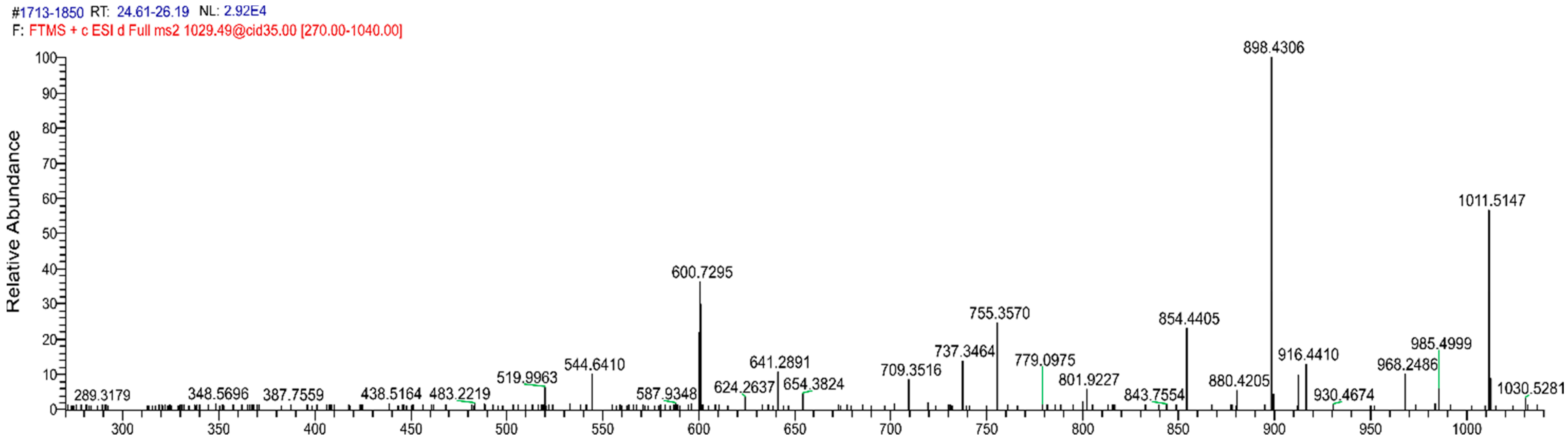

Figure S4. High resolution MS/MS spectrum of the $[M+H]^+$ ion at *m*/*z* 1029.5084, putatively identified as MC-M(O)R.

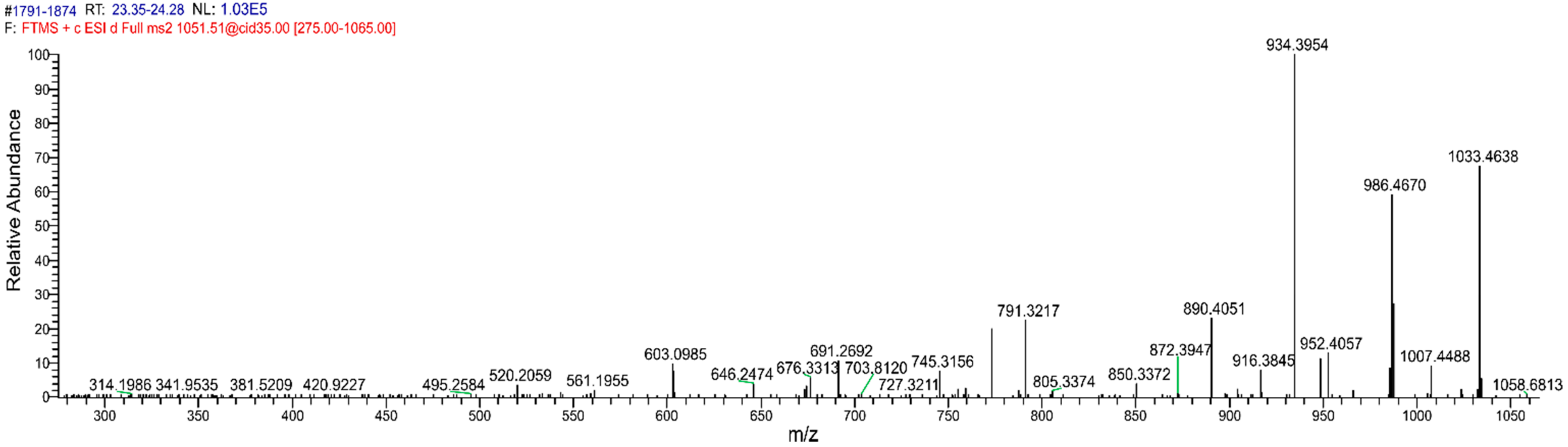

Figure S5. High resolution MS/MS spectrum of the $[M+H]^+$ ion at *m/z* 1051.5120, putatively identified as MP-T1.

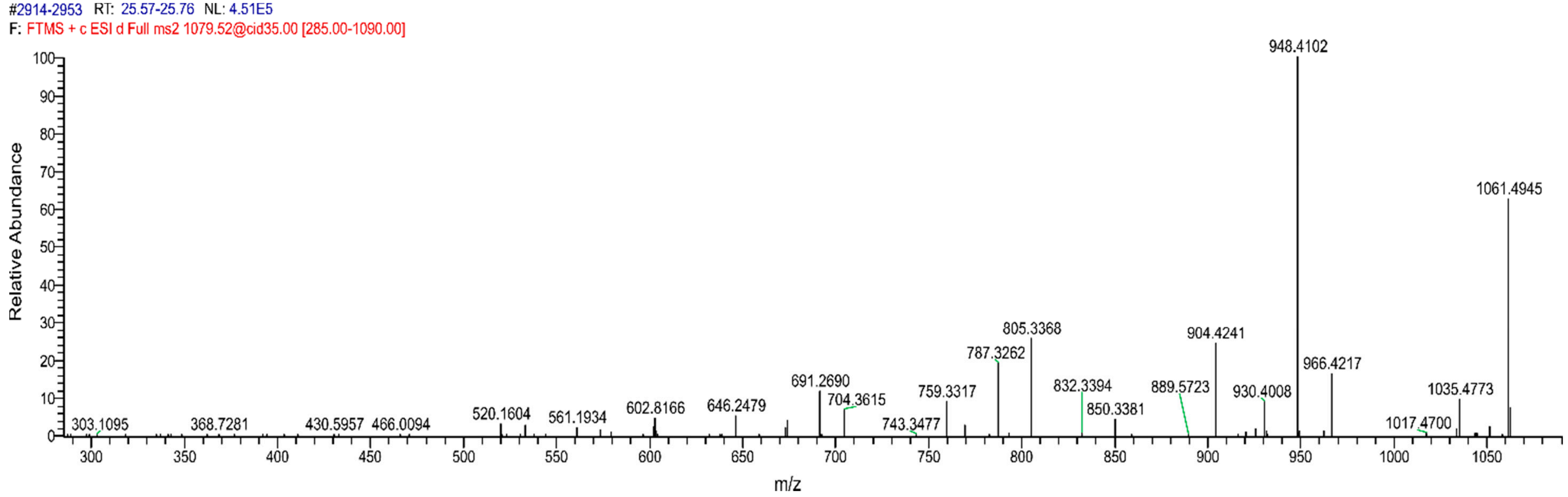

Figure S6. High resolution MS/MS spectrum of the $[M+H]^+$ ion at *m/z* 1079.5288, putatively identified as MP-88B.

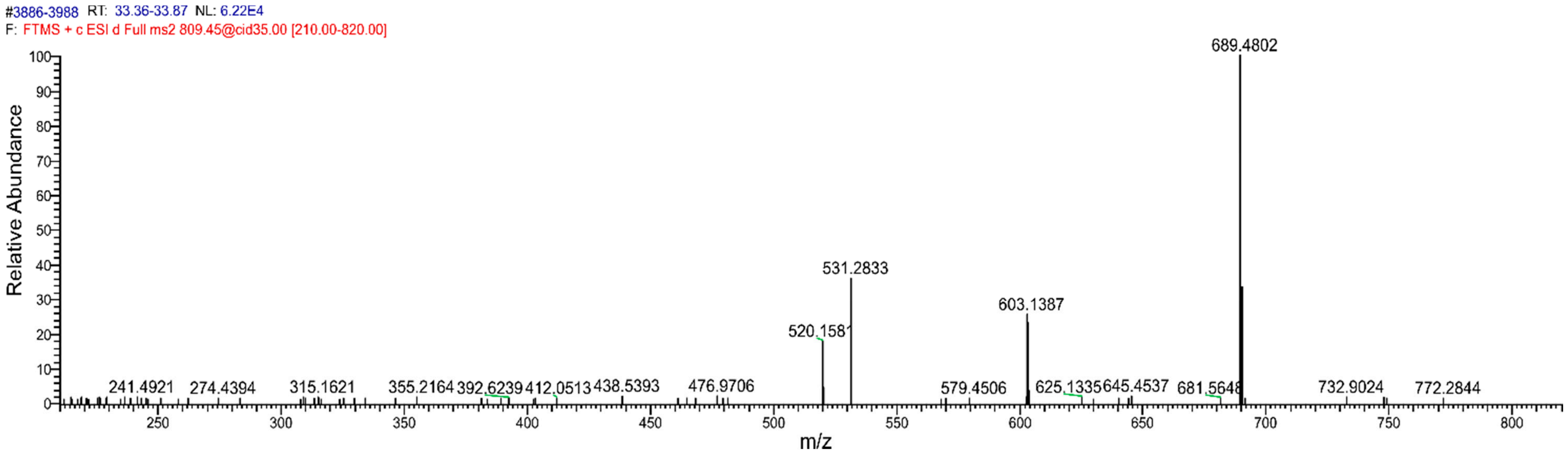

Figure S7. High resolution MS/MS spectrum of the [M+H]+ ion at *m/z* 809.4596, putatively identified as AnaP-C.

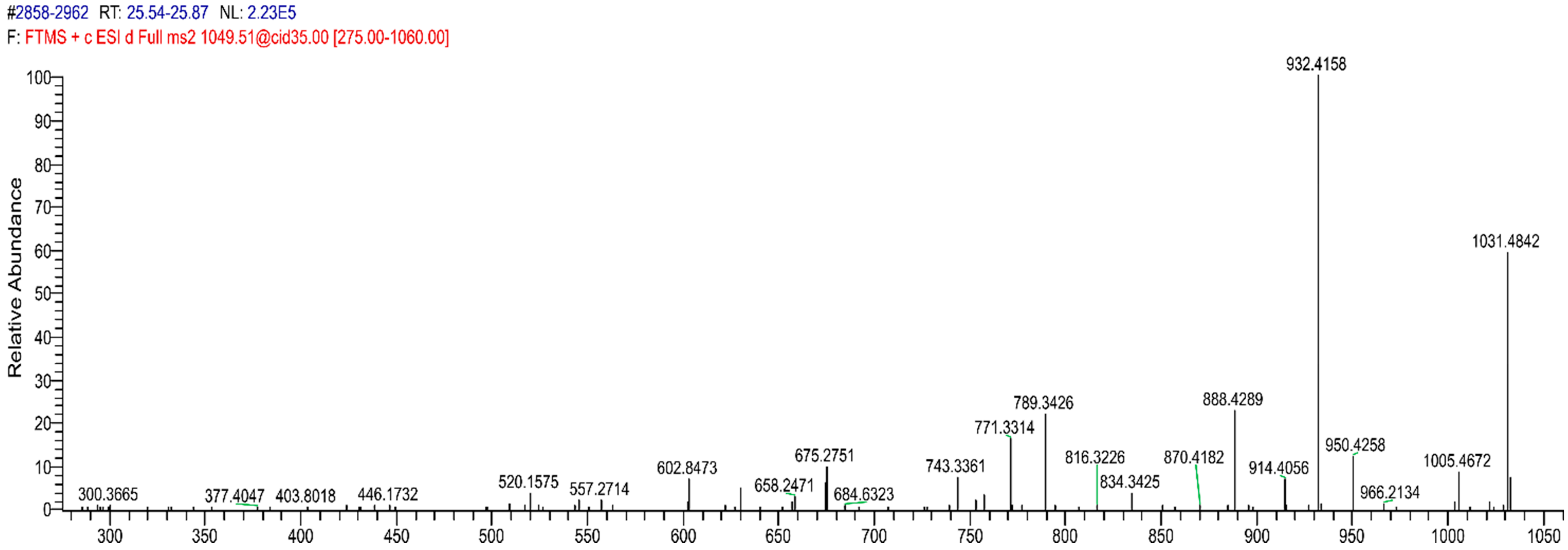

Figure S8. High resolution MS/MS spectrum of the [M+H]+ ion at *m/z* 1049.5165, putatively identified as AerP-228B.
